# From Research to Reality: Minimizing the Effects of Hospitalization on Older Adults

**DOI:** 10.5041/RMMJ.10201

**Published:** 2015-04-29

**Authors:** Hanna Admi, Efrat Shadmi, Hagar Baruch, Anna Zisberg

**Affiliations:** 1 Nursing Directorate, Rambam Health Care Campus, Haifa, Israel;; 2 Cheryl Spencer Department of Nursing and Faculty of Social Welfare and Health Sciences, University of Haifa, Mount Carmel, Israel

**Keywords:** Functional decline, geriatric nursing, hospitalization, older adults

## Abstract

This review examines ways to decrease preventable effects of hospitalization on older adults in acute care medical (non-geriatric) units, with a focus on the Israeli experience at the Rambam Health Care Campus, a large tertiary care hospital in northern Israel. Hospitalization of older adults is often followed by an irreversible decline in functional status affecting their quality of life and well-being after discharge. Functional decline is often related to avoidable effects of in-hospital procedures not caused by the patient’s acute disease. In this article we review the literature relating to the recognized effects of hospitalization on older adults, pre-hospitalization risk factors, and intervention models for hospitalized older adults. In addition, this article describes an Israeli comprehensive research study, the Hospitalization Process Effects on Functional Outcomes and Recovery (HoPE-FOR), and outlines the design of a combined intervention model being implemented at the Rambam Health Care Campus. The majority of the reviewed studies identified preadmission personal risk factors and psychosocial risk factors. In-hospital restricted mobility, under-nutrition care, over-use of continence devices, polypharmacy, and environmental factors were also identified as avoidable processes. Israeli research supported the findings that preadmission risk factors together with in-hospital processes account for functional decline. Different models of care have been developed to maintain functional status. Much can be achieved by interdisciplinary teams oriented to the needs of hospitalized elderly in making an impact on hospital processes and continuity of care. It is the responsibility of health care policy-makers, managers, clinicians, and researchers to pursue effective interventions to reduce preventable hospitalization-associated disability.

## INTRODUCTION

Over the past century the average human lifespan has doubled. The global share of older people (aged 60 years or over) increased from 9.2% in 1990 to 11.7% in 2013 and will continue to grow as a proportion of the world population, reaching 21.1% by 2050. Globally, the number of older persons is expected to more than double within the next several decades, from 841 million people in 2013 to more than 2 billion in 2050. By 2047, the number of older persons is projected to exceed the number of children for the first time. While people are living longer almost everywhere, the prevalence of noncommunicable diseases and disabilities is increasing as the population ages. This human transformation is creating new challenges in almost every aspect of life (social, political, and economic) with implications for health care, which have never before existed for our species.^1^–^3^

Older adults are considered vulnerable members of society with regard to their health, and the number of hospitalized elderly has been steadily increasing worldwide.^4^ Older adults constitute a major population in need of inpatient hospital services.^5^ Forty percent of hospitalized persons in the United States are 65 years of age or older.^6^

Israel is one of the top 10 leading countries in the world with regard to life expectancy. In 2012, the proportion of those older than 65 years in Israel was 10.4%, representing double the 1960 rate of 5.0%. Thirty-four percent of all hospitalized patients in Israel in 2012 were 65 years of age or older. Most of them (50%–60%) were hospitalized in medical units and intensive care units (respiratory and cardiac), and in surgical, vascular, and eye units.^7^

Medicine and allied health professions devote significant efforts to improve the quality of life of older adults. A major force affecting personal well-being is the ability to function independently. Functioning is one of the most measured outcomes in medical studies and in nursing and other health professions.^8^–^10^ As people get older, a major goal of health care is to extend the years in which the person reports a high quality of life with minimal disabilities (disability-free life expectancy).^11^

A wide array of factors have been identified as contributing to functional disability in older age.^12^,^13^ One of the most significant of these factors is acute hospitalization.^14^,^15^ Some patients are admitted to the hospital with a diagnosis not directly leading to functional deterioration (e.g. pneumonia, urinary tract infection), yet they demonstrate a general decline in function after a hospital stay.^16^–^18^ Recent literature reviews show that functional decline is one of the most common negative outcomes of hospitalization, with far-reaching consequences^19^,^20^ for the patient, family, and health care system.^21^

The finding of negative hospitalization outcomes in older adults was described as early as the 1980s. McVey and colleagues^22^ reported that about 30% of older acute adults developed additional disabilities in activities of daily living (ADL) while hospitalized. A high incidence of new disabilities in older adults was demonstrated in a 1982 survey among 279 patients at a community hospital. More than half of patients aged 75 and older needed assistance in ADL after hospitalization.^23^

A decade later, a prospective study among 1,279 patients in acute medical wards of five hospitals in the United States showed that 32% experienced functional decline in ADL, and 41% experienced a decrease in instrumental ADL (IADL) compared to two weeks prior to hospitalization. Only half of those who experienced post-hospitalization functional decline returned to their basic functioning at three months after discharge.^24^ Others have reported the development of new functional impairment among 60% of older hospitalized patients.^25^,^26^

More recent studies support these early findings, showing that patients aged 65 and older often suffer from functional decline during and after hospitalization.^16^,^27^–^29^ Post-hospitalization functional decline has been shown to be sustained up to one year following discharge, and non-recovery to baseline functional status has been associated with increased risk of institutionalization, prolonged disability, and death (up to three years).^15^

To address this phenomenon and examine the risk factors related to post-hospitalization functional decline as well as which interventions may help to prevent functional decline of older hospitalized adults, we performed a comprehensive review of the literature. In addition, we specifically focused on the Israeli experience based at the Rambam Health Care Campus, a large tertiary care hospital in northern Israel.

## RISK FACTORS FOR FUNCTIONAL DECLINE

Personal, psychosocial, and in-hospital factors are considered potential risk factors affecting the health and well-being of older adults. It is important to identify these risk factors in order to identify patient groups in need of preventive interventions.

### Personal Risk Factors

Personal risk factors have been suggested to play an important role in mortality, disability, and morbidity related to hospitalization. Most research focuses on factors that enable the identification of populations at risk.^16^,^30^,^31^ A literature review by Hoogerduijn et al.^32^ identified the following factors: age, low ADL or Instrumental Activity of Daily Life (IADL) prior to hospitalization, and low cognitive status. These factors were found to be significant predictors in the majority of studies evaluating functional decline during hospitalization, and they remained significant after controlling for severity of acute illness and co-morbidity scores. Interestingly, severity of illness and co-morbidities as well as primary diagnoses were not associated with functional decline in most studies that focused on non-disabling primary diagnoses while controlling for intervening factors.^30^,^33^ Some studies also identified nutritional status and malnutrition risk as major contributors to post-hospital functional decline.^34^,^35^

### Psychological Risk Factors

The hospital setting may also exert negative psychological consequences, causing older patients to feel worthless, fearful, or not in control of what is happening to them.^36^ Depressive symptoms among older adults are common and have been considered to represent a silent epidemic of modern life, and hospitalization has been identified as a potential risk factor for depressive symptoms in older adults.^34^ Indeed, depressive symptoms are a common phenomenon among older hospitalized patients.^37^ Of the 684 participants in a study that examined the hospitalization process effects on functional outcomes and recovery (HoPE-FOR), 13.5% expressed severe depression, another 18.4% moderate depression symptoms, and 18.7% suffered from anxiety.^38^ In many cases, the depressive symptoms related to hospitalization did not remit at discharge, and it was reported that more than 40% continued with these symptoms at three months post-discharge.^39^ Given the fact that affective disorders are often under-diagnosed, it is not clear how much of the emotional burden is due to a pre-existing condition or represents a consequence of acute illness and hospitalization.

### Social Factors

Social support during hospitalization is most prominently expressed through the involvement of informal caregivers. In Israel it has been found that 69%–78% of patients report having family members who assist them during their hospital stay.^40^,^41^ Informal caregivers are an important resource for patients during hospitalization; they act as a bridge between the patients’ environment and the unfamiliar hospital setting.^42^–^44^ In many cases, the family member is an integral part of the care team and performs some of the basic care activities.^43^,^45^,^46^ Yet, studies that examined the impact of informal caregivers on diverse hospitalization outcomes have shown inconclusive results. While some studies demonstrated a positive effect of family caregivers on a patient’s cognitive status,^47^ depression symptoms,^41^ and compliance to care,^48^ others demonstrated inconclusive or even negative outcomes related to delirium incidence, length of stay, or rehospitalization.^49^–^51^

### In-hospital Factors

Recently, the study of possible reasons for functional deterioration post-hospitalization has been gaining increased attention. Researchers, policy-makers, and practitioners seek to understand why, in some cases, as a health condition is cured or controlled, a new problem emerges, namely that of functional disability. In an attempt to discern the role of risk factors for in-hospital functional deterioration that may be amenable to change, the following in-hospital conditions have been the focus of recent research: restricted mobility, under-nutrition, continence, polypharmacy, and environmental factors.

*Restricted mobility* is often related to bed rest orders, limited access to bedside chairs, high beds, and the use of restraints, including intravenous poles and urinary catheters.^19^ Previous research indicated that 73%–83% of the measured hospital stay of older patients is spent lying in bed.^52^,^53^ Mobility has been shown to be directly related to immediate and long-term post-discharge functional outcomes such as ADL decline, new institutionalization, and death, when controlling for confounders.^54^,^55^

In-hospital *under-nutrition* and malnutrition is often a result of prolonged periods of orders restricting oral food intake, a diet not consistent with patient preferences, and the lack of access to food and fluids. Malnutrition has been shown to be associated with functional decline at discharge from hospital.^19^,^34^,^35^,^56^

*Continence* has been described as an important contributor to functional outcomes. One of the reasons for the high rates of hospital-acquired incontinence is the extensive use of specialized devices for frail, confused, or immobile patients, such as adult diapers and urinary catheters. Sometimes it is easier or more feasible to offer temporary devices rather than to exert effort on conservative and behavioral interventions such as habitual voiding strategies. The use of devices has been linked to deleterious effects such as pressure sores, recurrent urinary tract infections, depression, and functional dependency.^57^

*Polypharmacy* is common in older people. Although the purpose of drug therapy is therapeutic, medications are frequently associated with adverse outcomes, such as falls.^58^ Adverse outcomes related to medications occur 2.5 times more frequently in elderly than in patients under the age of 65. In a literature review by Hammond and Wilson^58^ polypharmacy in the elderly was associated with falls, but there was stronger evidence of association to the type of drug rather than to polypharmacy itself.^59^ Psychoactive drugs may be given to encourage sleep and sedation, despite the known possible adverse effect of these drugs on the elderly, as they increase the risk for falling, injury, and delirium.^60^–^62^ Benzodiazepine use has also been shown to affect post-hospitalization outcomes. Patients initiating these drugs in hospital are more likely to continue using them long-term, compared to controls.^63^

The human and physical *hospital environment* is often not safe, and tends to be a noisy, sensory and socially deprived, and disorienting environment. These environmental factors discourage mobility, exacerbate disorientation, disrupt sleep, lead to social isolation, and increase the likelihood of falls.^19^,^60^,^64^

In order to minimize avoidable effects of hospitalization-related processes on elderly patients, hospital managers and clinicians should adopt models of care that maintain the functional status of the hospitalized elderly and are tailored to the special needs of this frail population.

## MODELS OF INTERVENTIONS FOR HOSPITALIZED ELDERS

Different models of care have been developed to reduce the adverse effects of hospitalization (Table 1). Early evaluation and identification of elderly patients at risk provides the basis for prevention, for tailored treatment, and for the allocation of resources needed for interdisciplinary team care.^85^,^86^ The greatest benefit of evaluation and management has been shown for the most high-risk older adults.^87^,^88^ As a result of their continued contact with their patients and direct involvement in their care, nurses can supervise treatment and teach patients and families about care, and therefore they play an essential role in this process.^32^

**Table 1.** Summary of Intervention Models.

**Model**	**Purpose**	**Intervention**	**Findings**	**Limitations**
**NICHE^65^–^69^**	To improve outcomes for the elderly	Providing nurses with resources and guidance to improve care of hospitalized older adults	Enhanced knowledge and improved attitudes of nurses regarding incontinence, and reduced use of physical restraints	Mediated intervention with no guarantee of permanent change
**ACE^70^–^74^**	To provide holistic care for the elderly	Specially designed environment Patient-centered care Planned discharge by a multidisciplinary team Review of medical care	Lower risk of being discharged to nursing homes Less need for medical devices Lower incidence of depression Reported increased satisfaction with ACE unit as compared with standard care	Specialized ACE units more costly than regular units Need for resource allocation and specialized staff
**HELP^75^–^77^**	To retain physical and cognitive function; maximize independence at discharge; assist with hospital-to-home transition; and prevent unplanned readmissions	Daily volunteer visits Treatment by an interdisciplinary geriatric team	Reduced incidences of delirium, falls, and cognitive decline Cost-effective for many hospitals	Limited availability within only the dedicated department
**FFC^78^,^79^**	To assist nurses in teaching patients to help themselves in activities	Providing nurses with resources and guidance to support patient independence and physical activity	Nursing care that supports patient independence and physical activity may decrease functional deterioration	Requires further empirical testing of feasibility and potential outcomes
**CGA^80^–^84^**	To provide extensive geriatric evaluation	Personalized evaluation	Six and eight months after admission: lower mortality rate for patients assisted by this approach than for those not participating	Found no significant effects on physical function, re-hospitalization, or length of hospitalization
**Rambam Combined Model**	To improve treatment and outcomes for the elderly	Comprehensive geriatric evaluation Daily volunteer visits Providing nurses with guidance to improve care of hospitalized older adults	Data still being analyzed	Data still being analyzed

Most of the models focus on improving the quality of care, on preventing physical and cognitive deterioration, and on meeting the special needs of the elderly, rather than focusing on specific diseases.^89^ The most important aims of these models are to maintain functional and cognitive independence and thus reduce the need for post-discharge institutionalization, which is a major determinant of quality of life, cost, and prognosis.^87^

There are several models and intervention programs designed for older adults in the acute care setting.

### The Nurses Improving Care for Health System Elders Program

The aim of the Nurses Improving Care for Health System Elders (NICHE) program, developed in 1992 at the New York University, is to improve outcomes for the elderly. It focuses on nursing staff perceptions of the care environment for geriatric practice.^65^ The program provides nurses with resources and guidance to improve the care of hospitalized older adults. At its core, the program model calls for a change in organizational culture by placing elderly patients at the center of practice.^66^ Within this framework, each hospital unit identifies a number of nurses to receive geriatric education with emphasis on the major geriatric syndromes and their assessment and management. Geriatric Resource Nurses (GRN) receive ongoing updates in geriatrics, implement best clinical guidelines and protocols of geriatric care in their units, and become a source of information and guidance to other nurses. The GRNs may function in any unit that serves older adults, and, according to the proposed model, need to obtain support from advanced nurse practitioners in the field of gerontology.^67^

The main categories of the geriatric acute care program include guiding principles, leadership, organizational structures, physical environment, patient and family-centered approaches, aging-sensitive practices, geriatric staff competence, and interdisciplinary resources and processes.^68^ Implementation of the GRN evidence-based model requires hospitals to become members of NICHE, which serves as the technical resource and catalyst for networking among facilities committed to quality geriatric care.^65^ In organizations where the plan has been implemented, values such as respecting the rights of the elderly and including elderly patients in decision-making were incorporated into the organizational culture. A program evaluation study found improvement in knowledge and attitudes of nurses related to incontinence and reduction of physical restraint use,^69^ as well as improvement in documentation of geriatric syndromes, family support, and more positive attitudes toward pressure sore prevention. Unfortunately, to the best of our knowledge, no study has been conducted so far to evaluate this initiative’s influence on outcomes such as ADL functioning.

### The Acute Care of Elders Model

The Acute Care of Elders (ACE) model was developed in the 1990s and is based on holistic care rather than focusing mainly on medical diagnoses. This model offers change in four areas: a specially designed environment, patient-centered care, planned discharge by a multidisciplinary team, and review of medical care.^70^ The environment of a designated ACE unit is designed to encourage independent performance, and includes features such as carpeting in rooms and hallways, handrails, large clocks, large calendars, elevated toilet seats, and a parlor room.

Patient-centered care includes daily assessment of physical, cognitive, and psychosocial function. The aim of care is to prevent immobility, ADL dependence, malnutrition, falling, depression, and delirium.^71^ Discharge planning is interdisciplinary and aims at assisting patients to return home rather than to nursing homes, as soon as possible. Medical care is reviewed to prevent polypharmacy and medical procedure complications.

Elderly patients hospitalized in specialized ACE units had a better chance of benefitting from improved patient care and well-being in many aspects. It was found that these patients had a lower risk of being discharged to nursing homes, had less need for medical devices, had a lower incidence of depression, and reported increased satisfaction with the ACE unit compared to standard care.^72^,^73^ The costs of specialized ACE units were higher than regular units, but since the number of hospitalization days was reduced, the final cost was not significantly higher.^72^,^73^ Nevertheless, due to the need for resource allocation and specialized staff, and the limited number of hospital beds available on these units, many patients for whom an ACE unit would have been beneficial had to be hospitalized in regular units.^74^

### The Hospitalized Elder Life Program

The Hospitalized Elder Life Program (HELP) was developed at Yale University in 1999. This program was aimed at retaining physical and cognitive function in the elderly during hospitalization, to maximize independence at discharge, to assist in transition from hospital to home, and to prevent unplanned readmissions. In this program each patient is offered screening for six major risk factors related to cognitive decline and delirium: cognitive function, sleep deprivation, immobility, dehydration, vision, and hearing impairment. Patients with these risk factors are advised to receive a treatment protocol aimed at preventing further deterioration.^75^ The HELP protocols include a daily visitor program, therapeutic activities, early mobilization planning, non-pharmacologic sleep protocol, the use of hearing aids and glasses, treatment by an interdisciplinary geriatric team, and referral to community services. The interdisciplinary team included a geriatric nurse specialist, a geriatric physician, and a co-ordinator for additional services such as a dietitian, pharmacist, rehabilitation specialist, social worker, and trained volunteers. All team members aimed at reducing further patient deterioration.

In a patient and family satisfaction survey a high level of satisfaction was reported with the volunteers and nurses involved in the program.^76^ This program has been proven to lead to a reduction in the incidence of delirium, falls, and cognitive decline, as well as being cost-effective for many hospitals.^77^

### Function-focused Care

Function-focused care (FFC) is a philosophy of care in which nurses help patients to help themselves in activities such as eating, taking medications, washing, dressing, and mobilizing. For instance, the nurse will encourage the patient to walk to the washroom instead of letting him or her stay in bed and use a bedpan or wear a diaper.^78^,^79^ In a pilot study examining the effects of implementation of FFC for patients over the age of 70 hospitalized in an acute care setting, less functional deterioration was noted in these patients than in patients on units where FFC was not used. These results suggest that nursing care that supports independence and physical activity may decrease functional deterioration associated with hospitalization. In addition, there is a need to teach patients and their family members to encourage as much physical activity as possible, especially during hospitalization. Conclusions of the study suggest that it is especially important to pay attention to older adults admitted to hospital with pre-existing disabilities and to maintain their pre-hospital functional status.^78^ This program is promising but requires further empirical testing of its feasibility and potential outcomes.

### Comprehensive Geriatric Assessment

Comprehensive Geriatric Assessment utilizes a geriatric team to perform extensive geriatric evaluation of hospitalized patients. Patients undergo a careful history and physical examination, as well as social, functional, nutritional, and neuropsychological evaluations.^80^ The team assesses the older patient and recommends appropriate treatment and management. This comprehensive geriatric evaluation is considered part of the treatment plan and is much more cost-effective than specialized units. The team has easy access to a large number of patients and can respond fairly quickly to their needs.

This approach is used in a number of mainly European countries with the aim of improving quality of care in elderly patients hospitalized on general hospital units.^81^ In a systematic study of the outcomes in patients receiving this service, no significant effects on physical function, rehospitalization, or length of hospitalization were found.^82^ However, in a follow-up study performed six and eight months later, the mortality rate for patients treated by this approach was lower than for those who did not receive a comprehensive geriatric assessment.^81^

A comprehensive geriatric assessment can be performed in a Mobile Acute Care of the Elderly unit (MACE). A MACE service consists of a multidisciplinary team that promotes reducing the risks of hospitalization, improving co-ordination with community medical services and discharge planning, and educating patients and their caregivers. The staff contacts community services in order to co-ordinate discharge plans and ensure help at home for the patient.^83^

A comparison study between two groups of patients aged 75 and older was conducted to study the effects of this type of service. One group received services while the second did not. A smaller risk of adverse events during hospitalization (falls, bed sores, urinary tract infections, etc.) and shorter hospitalization were reported for patients cared for by units offering this service; however, the risk of rehospitalization within 30 days was similar. There was no difference in the physical functioning of patients from both groups.^84^

## THE ISRAELI EXPERIENCE: RESEARCH AND PRACTICE

As described in the introduction, similar to worldwide trends, Israel too is faced with an increasing geriatric population. The efforts of Israeli health care professionals to minimize the risks of hospitalization in geriatric patients further contribute to the body of knowledge on this important and challenging topic.

### Research: HoPE-FOR Study

Despite the importance of the many in-hospital risk factors, a comprehensive model that accounts for a wide range of known personal risk factors, together with information on hospitalization processes (i.e. medications, nutrition, mobility, the hospitalization environment, and support for independence) has never before been empirically tested in Israel. In order to provide more insight and in an effort to close this gap in knowledge we conducted the Hospitalization Process Effects on Functional Outcomes and Recovery (HoPE-FOR) study.^38^ (This Israeli study was initiated and led by A.Z. and E.S. together with H.A. and a team of researchers from the University of Haifa.) The methodology included a quasi-experimental design combining prospective and retrospective data collection. This multicenter study was conducted in the Rambam and Carmel medical centers from February 2009 until August 2011. The HoPE-FOR cohort was derived from a population of older adults (aged 70+) admitted to eight general medical inpatient units of two medical centers in the two hospitals. The inclusion criterion was admission to the hospital unit directly from the emergency room due to a non-disabling diagnosis. Patients with a new diagnosis of stroke, in a coma, mechanically ventilated, or completely dependent in basic functions before admission were not eligible for participation in the original study. Of the 2,308 eligible participants, 1,153 were screened and 1,032 participated in the study. The final sample included 813 patients who successfully completed the inhospital data collection for evaluating discharge outcomes and 703 who completed a one-month follow-up evaluation of post-discharge outcomes.

After signing informed consent, participants were asked to complete an interviewer-administered questionnaire four to six times during their hospitalization. The baseline interview asked about functional status upon admission and two weeks before hospitalization (premorbid functional status), as well as nutritional status, sleep medication use, continence care at home, depressive symptoms at admission, and demographics. During hospitalization, participants were asked about the following inhospital processes occurring within the previous 24 hours: mobility levels, continence care, sleep medication use, and nutritional intake. At discharge and at one-month follow up (by telephone interview) participants were asked to rate their functional status. Additional information on study recruitment, data collection, and the study instruments is available elsewhere.^38^,^55^,^57^,^90^–^92^

In the HoPE-FOR study we found a similar percentage of patients declining in their function as compared with previous European and US studies.^55^ The HoPE-FOR study emphasizes the significant contribution of within-hospitalization functional changes in the overall acute care functional continuum. Patients who remained stable before and during hospitalization had the highest odds of maintaining their premorbid functional levels, even after preadmission functional decline.^91^

The main findings of the HoPE-FOR study are that in-hospital mobility (measured as the walking inside and outside the patients’ room relative to mostly lying in bed) and the type of continence care (the use of continence care aids, including diapers and catheters) are directly related to functional decline of hospitalized older adults. The study also showed that low in-hospital nutritional intake is directly associated with functional decline one month post-discharge. These results were evident after accounting for a wide range of patient-related risk factors, including preadmission function, cognitive and nutritional status, chronic co-morbidities, acute severity of illness, and depression.^91^

### Practice: The Combined Model

The geriatric unit at Rambam Health Care Campus was established in 2014 and consists of two geriatricians, a nurse, and a part-time social worker. The unit offers multidisciplinary consultation services to all hospital departments. Social workers from the general departments and physical therapists are active partners in this venture and participate in deliberations regarding patients and treatment plans. There is close collaboration with other paramedical services.

After studying the models described above, it was decided that apart from providing geriatric consultation to the general departments, a proactive intervention plan would be implemented for older patients at risk in order to prevent functional and cognitive decline. The plan was designed by the Director of the Geriatric Unit, Professor Tzvi Dwolatzky, and is based on the principles of the HELP program described above. Material available online from HELP to clinicians was accessed (http://www.hospitalelderlifeprogram.org/) and was translated and adapted for use in Israel, taking into account aspects of local health policy, medical practice, law, and culture.

Following an initial period of staff and volunteer training, the project has been initiated, and to date more than 300 older adults hospitalized on internal medicine units have received visits and care from volunteers.

The criteria used for choosing patients to receive care from volunteers relate to their cognitive, physical, and clinical status. Eligible patients are able to hold a coherent conversation with the volunteer and are able to get out of bed with the aid *only* of a volunteer. Patients in critical care or isolation are excluded from the program.

The program and its aims were presented to the nursing and medical staff of the participating departments, and patients agree to the help of volunteers. Demographic details of the patients are recorded. A statistical analysis of this information will be conducted in the future to examine the effects of the program on outcomes for hospitalized older adults.

## IMPLICATIONS FOR PATIENTS AND THE HEALTH CARE SYSTEM

The effect of acute care hospitalization on the elderly, as demonstrated in this review, particularly in acute care non-geriatric units, might be detrimental, and this is often unrelated to the cause of hospitalization. In order to minimize preventable adverse consequences, several issues should be taken into account with regard to older adults, their families, and the health care systems: pre-existing patient frailty including aging and chronic comorbidity; the severity and type of acute disease that led to hospitalization; in-hospital organizational processes such as the hospital’s vision and policy, structure, and quality of care; and finally, the continuity of care after discharge.^88^,^93^

### Implications for Patient and Family

Physical recuperation of older adults is more difficult and requires longer hospital stays; they often need to be released to nursing homes because of decreased functioning and the need for help. Almost 20% of hospitalized elderly will be readmitted within 30 days.^94^ Readmission has been found to be a significant factor in poorer outcomes of rehabilitation after hospitalization and in mortality in the year following discharge.^71^ Many older people need more help at discharge and often are not able to return home. A high percentage of hospitalized elderly discharged to nursing homes never return to their homes.^4^,^32^,^86^,^89^,^95^

With regard to the impact of functional decline on the hospitalized older person, many adverse irreversible consequences and complications have been well described in the literature, including falls, injury, pressure sores, recurrent urinary tract infections, delirium, change of housing, institutional arrangements, social isolation, morbidity, disability, and mortality.^19^,^60^–^62^,^64^

Family members are confronted with personal, emotional, and financial ramifications as the changes in functional status may force them into a new role as caregiver, or necessitate hiring help for their older family member.^71^ In addition, there are the increased costs of health care which, for this age group, are three times higher than for younger age groups.^64^

### Implications for the Health Care System

There is much the health care system can do to prevent and minimize the phenomenon of functional decline of hospitalized elderly people, particularly in the areas of health policy, clinical practice, education, and research.

Health policy at the governmental and organizational levels should start with a clear vision regarding the elderly. The aim should be to provide an environment that meets the special needs of the elderly, with quality tailored care to maintain functional status and continuity of care following discharge.^88^,^93^ An example of how health policy is shaped by governments and fiscal policy is demonstrated by Medicare’s health change policy for older persons in the USA. In response to financial federal strain, Medicare has been challenged to reduce costs, to implement environmental changes, and to find ways to achieve high-quality and safe care for hospitalized persons.^64^

Hospitals are designed to deliver high-technology, rapid, and effective acute care that is often not in accordance with the needs of frail older people. Hospital environments and processes are not designed to preserve and improve a patient’s functional status.^88^,^95^,^96^ Specialized geriatric units in an acute care hospital setting seem to be more effective in terms of meeting the needs of older hospitalized patients, minimizing functional deterioration, and cutting costs in comparison to general medical and surgical units.^97^ The continuity of care is essential and requires communication and efficient co-ordination, between the hospitals and the community, which does not always happen.^64^

The literature is replete with recommendations for clinical practice aimed at reducing functional decline. Early screening and assessment of potential problems and the recognition of at-risk patients provide a basis for planning clinical interventions. The physical structure and standards of care should be designed to prevent dependence.^88^ Recommendations include modification of the acute hospital physical environment (i.e. low beds without rails, carpeting, clocks, calendars, common eating area, and quiet environment).^71^ Interventions such as reality orientation and early proactive activities can be used to maintain or achieve optimal cognitive functional capacity.^97^

Actively facilitating ambulation by encouraging activities of daily living, physical activity, and self-care has proven to be effective in preventing avoidable functional decline in elderly hospitalized people.^55^,^78^ Special awareness of nutritional status should be raised, and withholding oral food and fluids should be kept to a minimum. Sensory stimulation can be increased by appropriate lighting and environmental design, the use of spectacles and hearing aids, and the provision of reading materials and recreation. Social activities, family participation, and a caring empathetic medical and nursing team all help to soften the rather harsh high-technology hospital environment.

Daily review of medications and engagement of clinical pharmacists is important to reduce polypharmacy and minimize use of psychoactive medication thus avoiding delirium. Discharge planning for home should start early and should include a co-ordinated approach involving the patient, family, physician, nurse, and social worker, together with other paramedicals.^19^,^95^ Interdisciplinary acute care teams play a key role in improving independence for older hospitalized patients. The relationships among physicians, nurses, other health professionals, and non-professional members on the team must reflect the importance of interdisciplinary care and the implementation of shared objectives.^98^

#### Implications for Geriatric Education

Provision of care for elderly patients in a hospital setting is often characterized by inadequate knowledge and awareness of special geriatric needs. Often the knowledge is not integrated into the daily practice of the health care team.^88^

Education specific to caring for geriatric clients should be part of the training programs for health care professionals and in-service training plans of hospitals.^97^

#### Implications for Research

Future research should examine the geriatric care attitudes and knowledge of the health care staff that provide care in non-geriatric units.^66^ Although a variety of interventions exist for the management of elderly hospitalized persons, few studies have examined the impact of the interventions using quality standardized measurements.^88^ Conroy et al.^5^ conducted a systematic bibliographic review of high-quality randomized controlled trials on comprehensive geriatric assessment for frail older people being *rapidly* discharged from acute hospital. The authors concluded that only a few such trials have been carried out and that their overall quality was poor. Well-designed intervention research studies are needed.^5^ Controlled intervention studies would be helpful to establish causality relations and examine the effectiveness of care modes for elders.^65^,^85^ Another shortcoming of research in this area is that long-term effects were not measured.^85^

## CONCLUSION

There is a wealth of accumulative evidence in the literature indicating that the hospitalization of older people leads to functional decline, disability, morbidity, and mortality that is not related directly to the cause of hospitalization. Many assessment tools and intervention models have been developed aiming at minimizing preventable in-hospital processes and environmental risk factors.

Health care professionals devote significant effort toward improving the quality of care of older people. However, acute care hospitals are designed to provide high-technology, effective, and rapid care that does not always conform to the needs of the frail elderly.

It is the responsibility of health care policy-makers, managers, clinicians, and researchers to continue searching for effective interventions to reduce preventable hospitalization-associated disability. The focus should start with the vision and attitudes of interdisciplinary teams suitable to geriatric needs; through changes in hospital processes affecting the physical and social environment; and end with continuity of care in the hospital–community interface targeted to improve functional status and quality of life.

## Abbreviations:

ACE: Acute Care of Elders;
ADL: activities of daily living;
FFC: Function-focused Care;
HELP: Hospitalized Elder Life Program;
IADL: instrumental activities of daily life;
MACE: Mobile Acute Care of the Elderly;
NICHE: Nurses Improving Care for Health System Elders.
